# Multidimensional machine learning for early neurological deterioration prediction in acute ischemic stroke

**DOI:** 10.3389/fmed.2026.1779519

**Published:** 2026-04-24

**Authors:** Wei Wang, Genchun Guo

**Affiliations:** 1 Shinhan University of Physical Education, Uijeongbu-si, Gyeonggi-do, Republic of Korea; 2 Department of Rehabilitation Medicine, Affiliated Hospital 6 of Nantong University (Yancheng Third People’s Hospital, the Affiliated Hospital of Jiangsu Medical College), Yancheng, China

**Keywords:** acute ischemic stroke, early neurological deterioration, machine learning, multidimensional, predictive model

## Abstract

**Objective:**

This study aimed to develop and validate a multidimensional clinical feature-based machine learning model for accurately predicting the risk of early neurological deterioration (END) in patients with acute ischemic stroke (AIS).

**Methods:**

A total of 338 AIS patients were randomly divided into a training set (*n* = 236) and a validation set (*n* = 102). Five core predictors were identified from multiple clinical and pathological indicators: admission National Institutes of Health Stroke Scale (NIHSS) score, admission blood glucose, infarct core volume, collateral circulation status, and neutrophil-to-lymphocyte ratio (NLR). In the training set, univariate analysis was first performed to screen prognosis-related factors. After variable compression via least absolute shrinkage and selection operator (LASSO) regression, multivariate logistic regression was employed to determine independent risk factors for poor prognosis. Using Python, three prediction models-Random Forest (RF), Gradient Boosting Machine (GBM), and K-Nearest Neighbors (KNN)-were constructed. Model performance was evaluated by the area under the receiver operating characteristic curve (AUC), and the optimal model was selected.

**Results:**

No statistically significant differences were observed in baseline characteristics between the training and validation sets (*P* > 0.05). Multivariate logistic regression revealed that admission NIHSS score, blood glucose, infarct core volume, and NLR were independent risk factors (*P* < 0.05), while collateral circulation status was an independent protective factor (*P* < 0.05). The RF model demonstrated superior predictive performance, with AUC values of 0.779 (training set) and 0.775 (validation set), significantly outperforming KNN (0.727, 0.741) and GBM (0.736, 0.665).

**Conclusion:**

The multidimensional model provides a potential practical tool for early clinical identification of high-risk END patients and timely intervention.

## Introduction

Acute ischemic stroke (AIS) is one of the leading causes of disability and mortality worldwide, imposing a substantial burden on society and families (1). Despite landmark advances in hyperacute revascularization therapies, the post-stroke disease course remains highly variable, with a significant proportion of patients at risk of clinical progression (2). Among these challenges, early neurological deterioration (END) represents a common yet formidable clinical complication following AIS (3). END typically refers to unexpected worsening or fluctuation in neurological deficits within the initial hours to days after stroke onset (4). Although its precise definition varies slightly across studies, a commonly adopted criterion is an increase of ≥ 2 or ≥ 4 points on the National Institutes of Health Stroke Scale (NIHSS). Extensive literature indicates that END occurs in 10–40% of patients and is strongly associated with poor long-term functional outcomes, prolonged hospitalization, and elevated mortality (5).

The pathophysiological mechanisms underlying END are complex and multifactorial, involving several key aspects: First, ischemic progression serves as the central mechanism, wherein thrombus extension or new emboli lead to the conversion of the ischemic penumbra into the infarct core, directly driving neurological decline (6). Second, secondary brain injury processes, such as excessive activation of post-ischemic inflammation, excitatory amino acid toxicity, and massive free radical production, collectively exacerbate blood-brain barrier disruption and neuronal death (7). Additionally, systemic factors, including fever, infection, dysglycemia, and hemodynamic instability, may contribute to END by reducing cerebral perfusion or increasing metabolic demand (8).

Currently, clinical practice lacks a unified and efficient tool for END prediction. Traditional approaches predominantly rely on physicians’ empirical judgment or isolated predictors, such as higher baseline NIHSS scores, admission hyperglycemia, or large vessel occlusion on imaging (9, 10). However, these individual indicators exhibit limited predictive performance, often failing to balance specificity and sensitivity, and inadequately capture the multifactorial nature of END. Therefore, there is an urgent need for a predictive model capable of integrating multidimensional data to quantify individual risk, enabling early identification of high-risk patients.

Advances in medical informatics have provided novel solutions to such complex challenges through machine learning (ML). ML algorithms can autonomously learn intricate nonlinear relationships and interactions from high-dimensional data, uncovering patterns beyond conventional statistical models (11). In stroke research, ML has been successfully applied to diagnostic classification, outcome prediction, and imaging analysis (12). A multidimensional ML model incorporating clinical assessments, neuroimaging, and serum biomarkers may theoretically provide a more comprehensive representation of END pathophysiology, thereby enabling more precise risk stratification.

Against this background, this study aimed to retrospectively collect clinical data from AIS patients, identify core predictors closely associated with END, and develop a robust predictive model using advanced ML algorithms. It is anticipated that this model will serve as an early and convenient decision-support tool for clinicians, facilitating timely intervention for high-risk patients and ultimately improving outcomes.

## Materials and methods

### Study population

This single-center, retrospective observational study consecutively screened AIS patients admitted to the Department of Neurology between June 2022 and June 2025. Inclusion criteria were: (1) age ≥ 18 years; (2) time from onset to admission < 24 h (13); (3) completion of baseline magnetic resonance imaging (including DWI and PWI sequences) and relevant laboratory tests upon admission. Exclusion criteria included: (1) receipt of intravenous thrombolysis or endovascular thrombectomy (to avoid reperfusion-related confounding); (2) death or discharge within 24 h of admission; (3) severe organ dysfunction or active infection; (4) incomplete clinical or imaging data. Ultimately, 338 patients were included in the analysis.

### Data collection

Data were collected using a predefined standardized case report form. All variables were independently extracted by two researchers from electronic medical records and cross-checked for accuracy. Demographic data (age, gender) and medical history (hypertension, diabetes, atrial fibrillation, prior stroke, or transient ischemic attack [TIA]) were recorded. Admission clinical features included NIHSS score (baseline), Glasgow Coma Scale (GCS) score, first-measured systolic blood pressure, and point-of-care glucose. Imaging data were analyzed by two blinded neuroradiologists unaware of clinical outcomes. MRI was performed using a 3.0T scanner (Siemens Skyra) with the following sequences: diffusion-weighted imaging (DWI, b-values 0 and 1,000 s/mm^2^, slice thickness 5 mm), perfusion-weighted imaging (PWI, dynamic susceptibility contrast, 20 time-points), and time-of-flight MR angiography. Infarct core volume was measured as the volume of the restricted diffusion area on DWI, calculated using semi-automated segmentation software (Olea Sphere, v3.0). Hypoperfusion volume was defined as the area with prolonged mean transit time (>145% of contralateral hemisphere) on PWI. Collateral circulation status was assessed on MR angiography using the modified Tan scale, with good collateral defined as collateral supply filling > 50% of the distal middle cerebral artery territory. Laboratory data consisted of the first venous blood test results post-admission. The neutrophil-to-lymphocyte ratio (NLR) was calculated by dividing absolute neutrophil count by absolute lymphocyte count from complete blood cell analysis. Inflammatory markers, such as C-reactive protein (CRP), were also documented.

### Outcome definition

The primary outcome of this study was END, operationally defined as an increase of ≥ 4 points in the total NIHSS score within 72 h of admission compared to baseline. To ensure consistency and accuracy in assessment, all NIHSS scores were independently evaluated by two attending neurologists (or higher-ranking physicians) who underwent standardized training and were blinded to other study data. Based on the outcome definition, patients were categorized into two groups. END group: Patients with an increase of ≥ 4 points in NIHSS score within 72 h of admission. Non-END group: Patients with an increase of < 4 points in NIHSS score within 72 h of admission.

### Sample size estimation

Sample size was determined using the events per variable (EPV) principle. Based on literature and clinical experience, the estimated incidence of END was 24%, and five candidate predictors were initially planned. With an EPV ≥ 5 considered acceptable for preliminary exploration, the minimum required events were 25, yielding a required sample size of 25/0.24 ≈ 104 patients. After accounting for a potential 20% data loss, the minimum enrollment target was set at 104/0.8 = 130 patients.

This target represented the minimum requirement for theoretical feasibility. However, given the exploratory nature of the study and the need to ensure adequate power for subsequent analyses, we enrolled all eligible patients consecutively admitted between June 2022 and June 2025 (*n* = 338). Patients were randomly divided into training (*n* = 236, 70%) and validation (*n* = 102, 30%) sets. In the training set, 57 END events occurred. The final model included five predictors, corresponding to an EPV of 11.4 (57/5), which substantially exceeds the EPV ≥ 5 criterion and confirms the model’s statistical robustness.

### Statistical analysis

Statistical analyses were performed using SPSS 26.0 and R 4.2.3. Normally distributed continuous variables were expressed as mean ± standard deviation (SD) and compared with Student’s *t*-test, while non-normally distributed data were presented as median (interquartile range) and analyzed using the Mann-Whitney U test. Categorical variables were reported as counts (percentages) and compared via χ^2^-test. In the training set, univariate analysis was first conducted to screen variables with *P* < 0.05. After variable compression by least absolute shrinkage and selection operator (LASSO) regression to filter out the most predictive core variables and avoid overfitting caused by excessive variables, multivariate logistic regression was further employed to quantify the independent predictive effect of each core variable on END and identify the independent risk factors and protective factors, and their odds ratios (OR) and 95% confidence intervals (CI) were calculated. Random Forest (RF), Gradient Boosting Machine (GBM), and K-Nearest Neighbors (KNN) models were constructed using Python 3.8.5 with the scikit-learn library. To mitigate potential bias due to class imbalance, the RF model was trained with the class_weight = “balanced” parameter to adjust class weights accordingly. To ensure robust evaluation of model stability, we additionally performed 5-fold cross-validation on the training set. Receiver operating characteristic (ROC) curves were plotted with GraphPad Prism 9.0. Model performance was further evaluated using accuracy, sensitivity, specificity, positive predictive value (PPV), and negative predictive value (NPV) at the optimal probability threshold determined by the Youden index. Calibration was assessed by plotting observed versus predicted probabilities (calibration curve) and quantified using the Brier score. SHapley Additive exPlanations (SHAP) analysis was applied to quantify the feature importance of core predictors and interpret the machine learning models, and based on the optimal model, the nomogram was developed. A *P* value < 0.05 was considered statistically significant.

## Results

### Comparison of baseline characteristics between training and validation sets

A total of 338 participants were enrolled and divided into a training set (*n* = 236, 70%) and a validation set (*n* = 102, 30%). No significant differences were observed in baseline characteristics between the two sets (*P* > 0.05) (Table 1).

**TABLE 1:** Comparison of baseline characteristics between training and validation sets.

Variables		Training set (*n* = 236)	Validation set (*n* = 102)	*t/χ* ^2^	*P*
Age (years)		67.81 ± 10.94	66.83 ± 11.89	0.736	0.462
Gender, n (%)	Male	142 (60.17)	56 (54.90)	0.814	0.367
	Female	94 (39.83)	46 (45.10)		
History of hypertension, n (%)	Yes	172 (72.88)	71 (69.61)	0.378	0.539
	No	64 (27.12)	31 (30.39)		
History of diabetes, n (%)	Yes	75 (31.78)	30 (29.41)	0.187	0.666
	No	161 (68.22)	72 (70.59)		
History of atrial fibrillation, n (%)	Yes	60 (25.42)	28 (27.45)	0.152	0.697
	No	176 (74.58)	74 (72.55)		
History of stroke/TIA, n (%)	Yes	54 (22.88)	21 (20.59)	0.217	0.641
	No	182 (77.12)	81 (79.41)		
Admission NIHSS score		10.23 ± 5.79	9.71 ± 5.94	0.752	0.453
Baseline impaired consciousness, n (%)	GCS < 15	68 (28.81)	27 (26.47)	0.194	0.660
	GCS ≥ 15	168 (71.19)	75 (73.53)		
Admission systolic blood pressure (mmHg)		153.11 ± 23.84	150.42 ± 26.07	0.925	0.355
Admission blood glucose (mmol/L)		7.94 ± 2.68	7.55 ± 2.43	1.262	0.208
Infarct core volume (mL)		28.51 ± 25.10	26.84 ± 24.31	0.567	0.571
Hypoperfusion volume (mL)		82.50 ± 52.03	78.91 ± 49.83	0.590	0.556
Large artery occlusion, n (%)	Yes	125 (52.97)	53 (51.96)	0.029	0.865
	No	111 (47.03)	49 (48.04)		
Collateral circulation, n (%)	Good	124 (52.54)	51 (50.00)	0.184	0.668
	Poor	112 (47.46)	51 (50.00)		
Neutrophil-to-lymphocyte ratio		5.49 ± 3.81	5.23 ± 3.60	0.585	0.559
C-reactive protein (mg/L)		8.14 ± 7.52	7.53 ± 6.79	0.704	0.482
Early neurological deterioration, n (%)	Deterioration	57 (24.15)	24 (23.53)	0.015	0.902
	No deterioration	179 (75.85)	78 (76.47)		

### Univariate analysis of risk factors for early neurological deterioration in acute ischemic stroke

Univariate analysis revealed statistically significant differences between the END and non-END groups in the training cohort for the following five parameters: admission NIHSS score, admission blood glucose, infarct core volume, collateral circulation status, and NLR (all *P* < 0.05) (Table 2).

**TABLE 2:** Univariate analysis of factors influencing early neurological deterioration in acute ischemic stroke.

Variables		END group (*n* = 57)	Non-END group (*n* = 179)	*t/χ* ^2^	*P*
Age (years)		68.93 ± 10.84	67.21 ± 11.12	1.023	0.307
Sex, n (%)	Male	33 (57.89)	109 (60.89)	0.162	0.687
	Female	24 (42.11)	70 (39.11)		
History of hypertension, n (%)	Yes	44 (77.19)	128 (71.51)	0.707	0.401
	No	13 (22.81)	51 (28.49)		
History of diabetes, n (%)	Yes	22 (38.60)	53 (29.61)	1.611	0.204
	No	35 (61.40)	126 (70.39)		
History of atrial fibrillation, n (%)	Yes	17 (29.82)	43 (24.02)	0.768	0.381
	No	40 (70.18)	136 (75.98)		
History of stroke/TIA, n (%)	Yes	15(26.32)	39(21.79)	0.502	0.479
	No	42(73.68)	140(78.21)		
Admission NIHSS score		11.82 ± 5.13	9.90 ± 4.17	2.857	0.005
Baseline impaired consciousness, n (%)	GCS < 15	22 (38.60)	46 (25.70)	3.507	0.061
	GCS ≥ 15	35 (61.40)	133 (74.30)		
Admission systolic blood pressure (mmHg)		156.23 ± 25.14	151.83 ± 23.49	1.211	0.227
Admission blood glucose (mmol/L)		8.92 ± 2.79	7.74 ± 2.31	3.188	0.002
Infarct core volume (ml)		38.90 ± 26.71	26.82 ± 21.33	3.494	0.001
Hypoperfusion volume (mL)		93.14 ± 55.27	78.54 ± 48.15	1.922	0.056
Large artery occlusion, n (%)	Yes	36 (63.16)	89 (49.72)	3.134	0.077
	No	21 (36.84)	90 (50.28)		
Collateral circulation, n (%)	Good	20 (35.09)	104 (58.10)	5.886	0.015
	Poor	37 (64.91)	75 (41.90)		
Neutrophil-to-lymphocyte ratio		6.45 ± 3.91	4.80 ± 3.21	3.200	0.002
C-reactive protein (mg/L)		11.83 ± 10.26	9.82 ± 7.04	1.667	0.097

NIHSS, National Institutes of Health Stroke Scale.

### LASSO regression for feature selection

**FIGURE 1:**
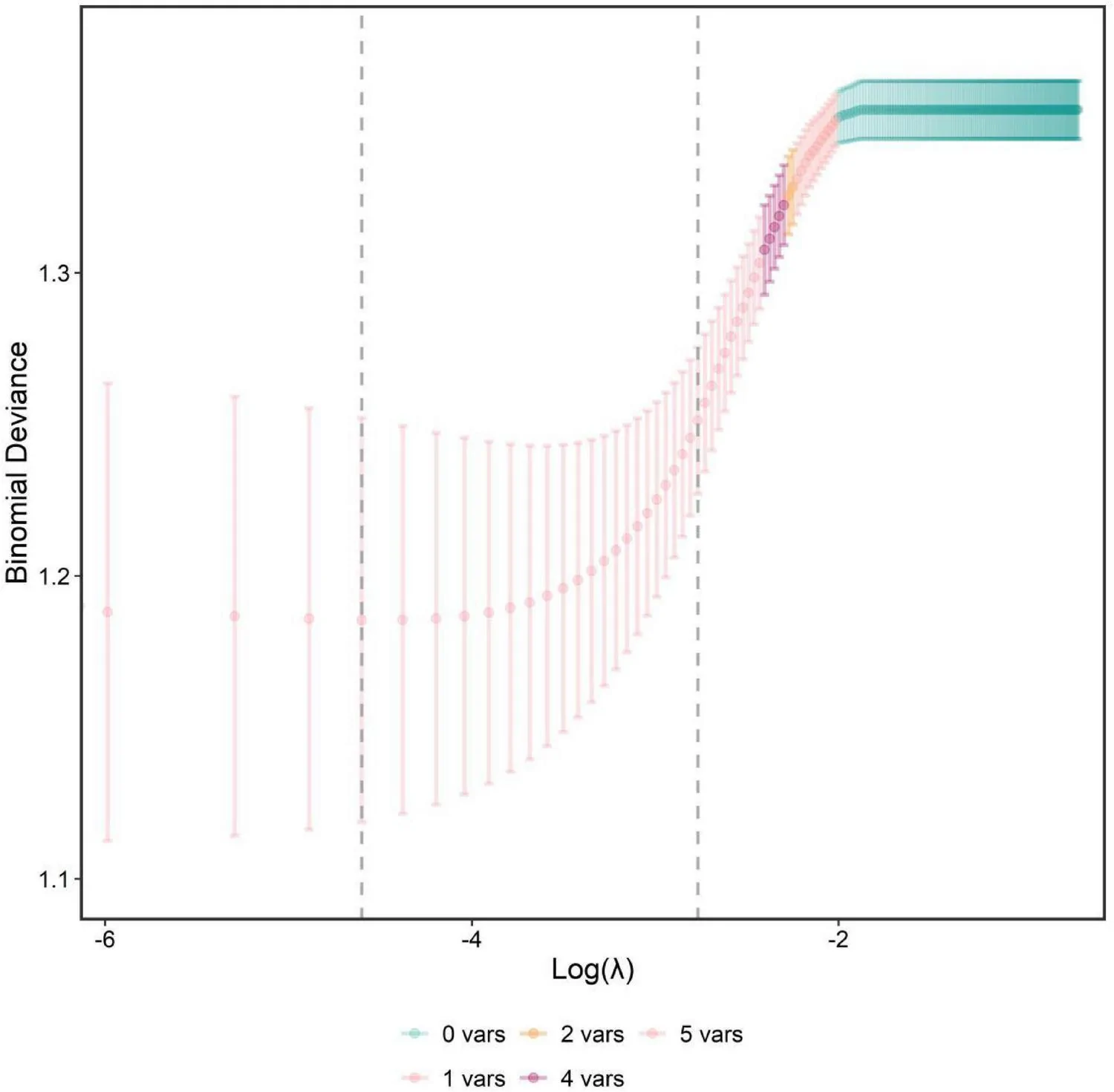
LASSO regression coefficient paths.

To identify the most predictive features for END while preventing overfitting, LASSO regression was employed for feature selection. Figure 1 illustrated the coefficient paths of candidate predictors as the regularization parameter λ (log-transformed) increased. At lower λ values, most features were retained due to higher model complexity. With increasing λ (i.e., stronger regularization), coefficients were progressively shrunk toward zero. At the optimal threshold, five features-admission NIHSS score, admission blood glucose, infarct core volume, collateral circulation status, and NLR-were retained, while others were excluded due to negligible predictive contributions.

### Multivariate logistic regression analysis of factors influencing early neurological deterioration

Early neurological deterioration was set as the dependent variable (0 = non-END group, 1 = END group). Five predictive variables were retained: admission NIHSS score, admission blood glucose, infarct core volume, collateral circulation status, and NLR. Multivariate logistic regression demonstrated that admission NIHSS score, admission blood glucose, infarct core volume, and NLR were independent risk factors for END (*P* < 0.05), while robust collateral circulation served as an independent protective factor (Table 3).

**TABLE 3:** Multivariate logistic regression analysis of factors influencing END.

Variables	β	SE	Wald	*P*	OR	95%CI
Admission NIHSS score	0.101	0.038	6.978	0.008	1.106	1.026–1.192
Admission blood glucose	0.186	0.071	6.793	0.009	1.204	1.047–1.384
Infarct core volume	0.026	0.007	12.443	0.001	1.027	1.012–1.042
Collateral circulation status	−0.870	0.343	6.421	0.011	0.419	0.214–0.821
NLR	0.171	0.050	11.934	0.001	1.187	1.077–1.308

NIHSS, National Institutes of Health Stroke Scale; NLR, Neutrophil-to-lymphocyte ratio. Assignment of independent variables (categorical variables): Collateral circulation status (Poor = 0, Good = 1).

### Predictive performance of models in training and validation sets

**FIGURE 2:**
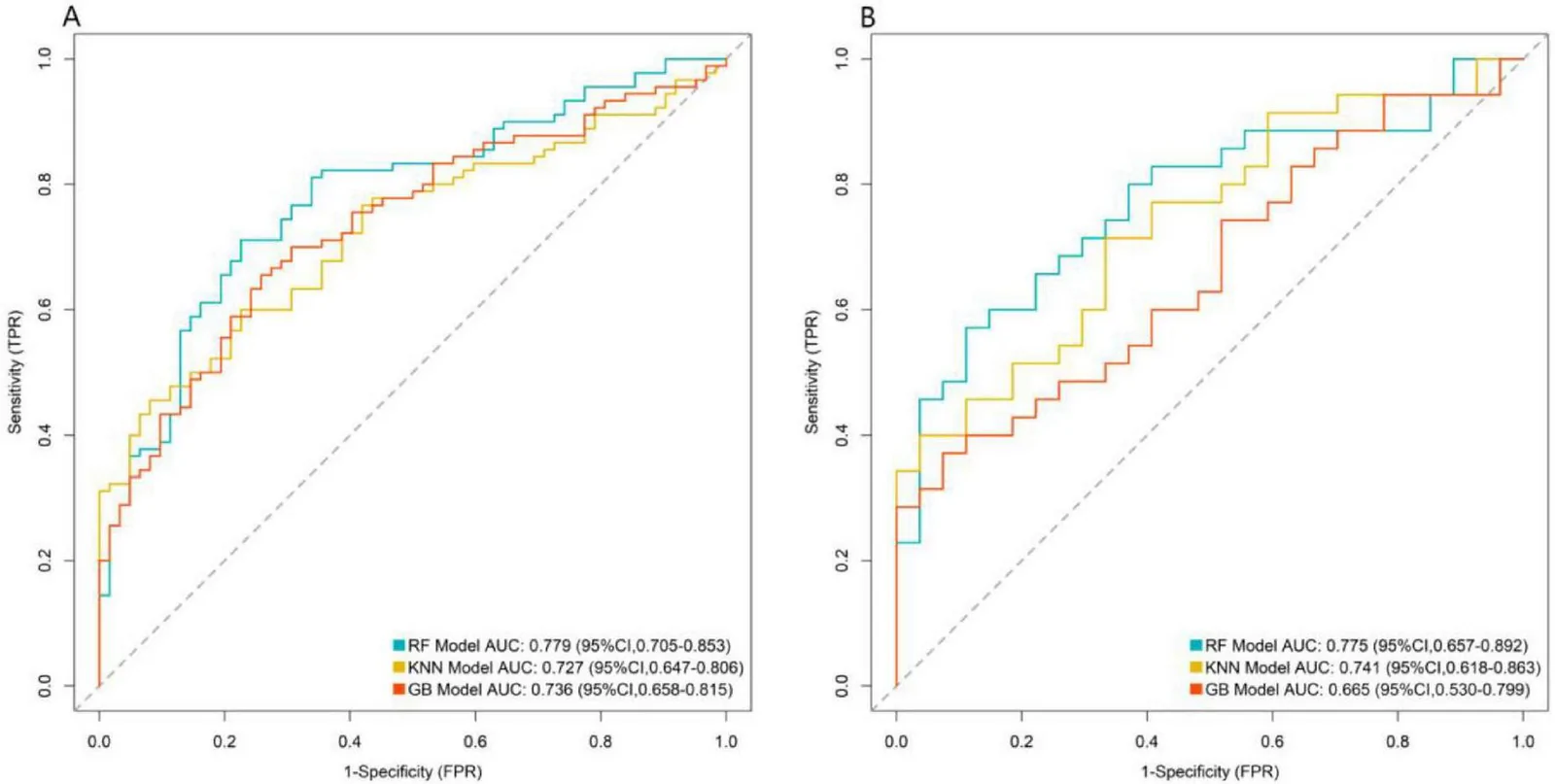
Receiver operating characteristic curves (**A:** training set; **B:** validation set). RF, Random Forest; GBM, Gradient Boosting Machine; and KNN, K-Nearest Neighbors.

**FIGURE 3:**
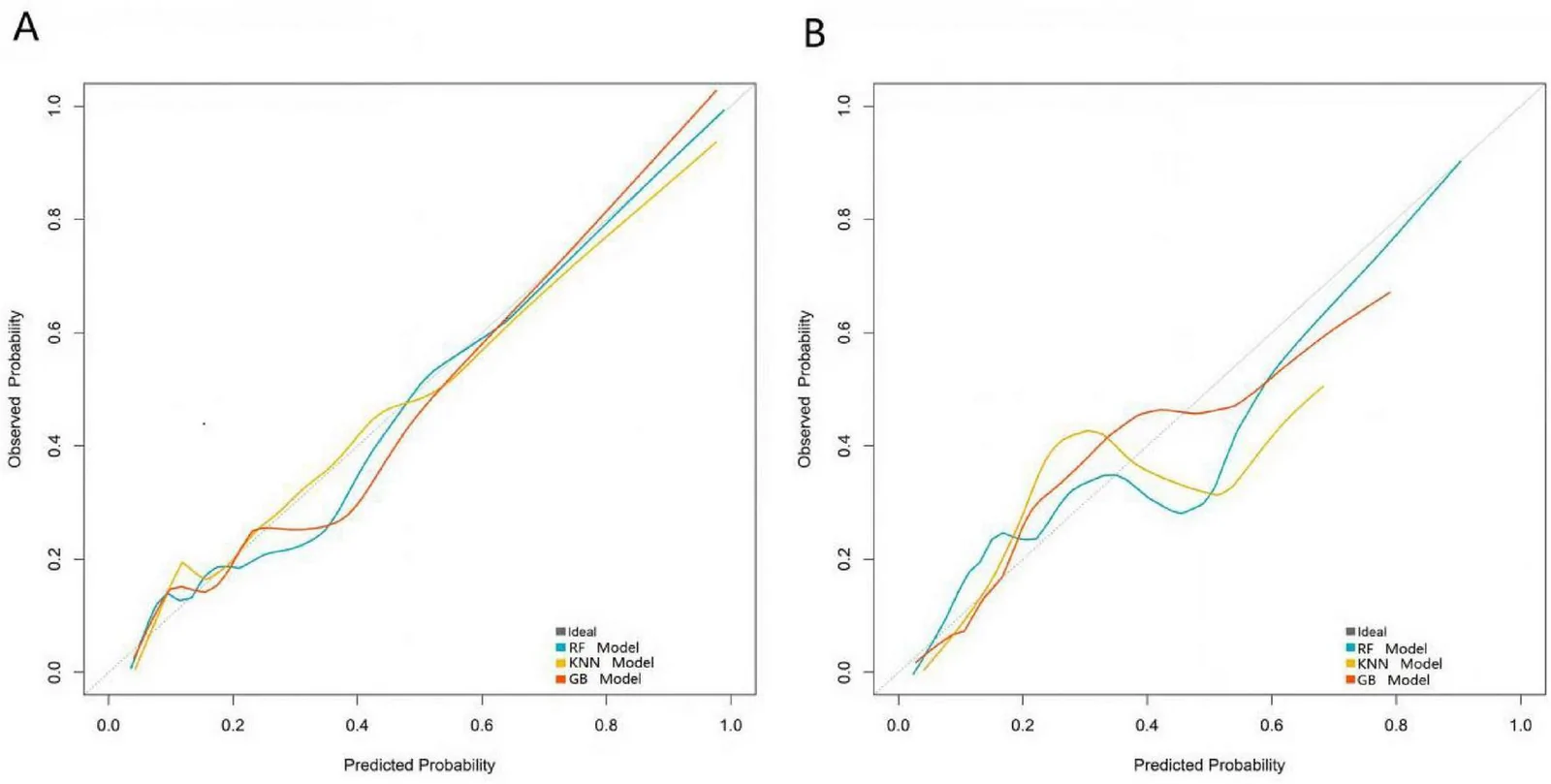
Calibration analysis of the prediction model in the training set **(A)** and validation set **(B).** RF, Random Forest; GBM, Gradient Boosting Machine; and KNN, K-Nearest Neighbors.

The RF, KNN, and GBM models were evaluated. In the training set, the AUC values were 0.779, 0.727, and 0.736, respectively; in the validation set, the corresponding AUC values were 0.775, 0.741, and 0.665. The 5-fold cross-validation results further confirmed the internal stability of the RF model, yielding a mean AUC of 0.772 ± 0.018 in the training set. The RF model, which achieved the highest AUC, was selected as the optimal predictive model (Figure 2). At the optimal probability threshold determined by the Youden index (0.26 in the training set, 0.25 in the validation set), the RF model achieved the following performance metrics in the training set: accuracy = 0.754, sensitivity = 0.702, specificity = 0.771, PPV = 0.494, and NPV = 0.891. In the validation set, the corresponding values were: accuracy = 0.735, sensitivity = 0.667, specificity = 0.756, PPV = 0.457, and NPV = 0.882. Calibration of the RF model was assessed by plotting observed versus predicted probabilities (Figure 3). The calibration curve lay close to the diagonal line, indicating good agreement between predicted and actual risks. The Brier score was 0.152 in the training set and 0.161 in the validation set, further confirming adequate prediction accuracy.

### Construction of the prediction model for early neurological deterioration

As the number of decision trees increased, the error stabilized, reflecting the dynamic performance of the model during iterative tree construction. This trend aided in assessing model convergence: when the error curve plateaued, further trees provided marginal improvement, guiding the selection of an optimal tree count to balance complexity and predictive accuracy (Supplementary Figure 1).

**FIGURE 4:**
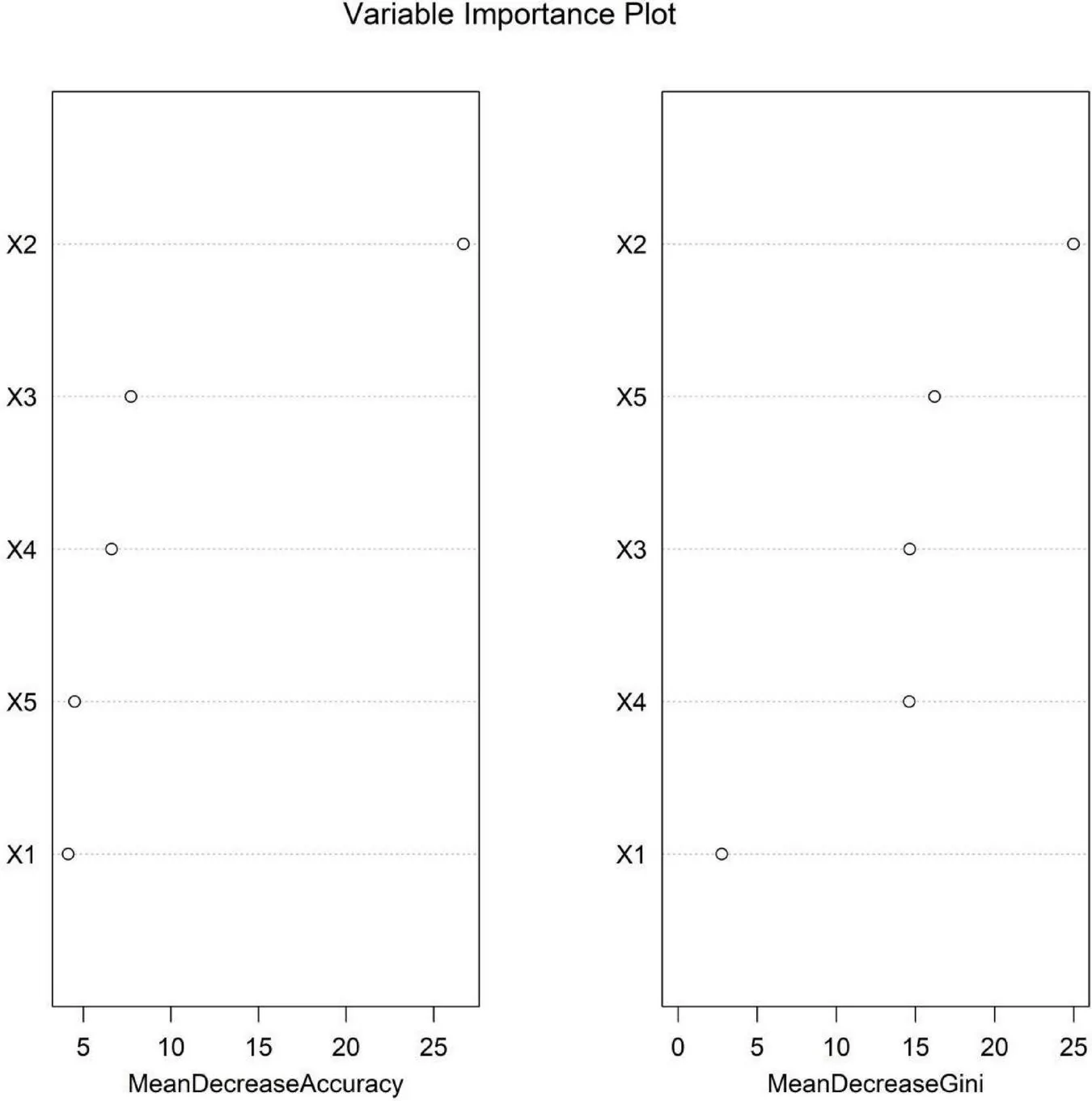
Feature importance ranking in the RF model. X1, Admission National Institutes of Health Stroke Scale score; X2, Admission blood glucose; X3, Infarct core volume; X4, Collateral circulation status; X5, Neutrophil-to-lymphocyte ratio.

The RF model ranked the independent predictors of END by importance scores in descending order: admission blood glucose, infarct core volume, collateral circulation status, NLR, and admission NIHSS score (Figure 4).

### Interpretability of model predictions

**FIGURE 5:**
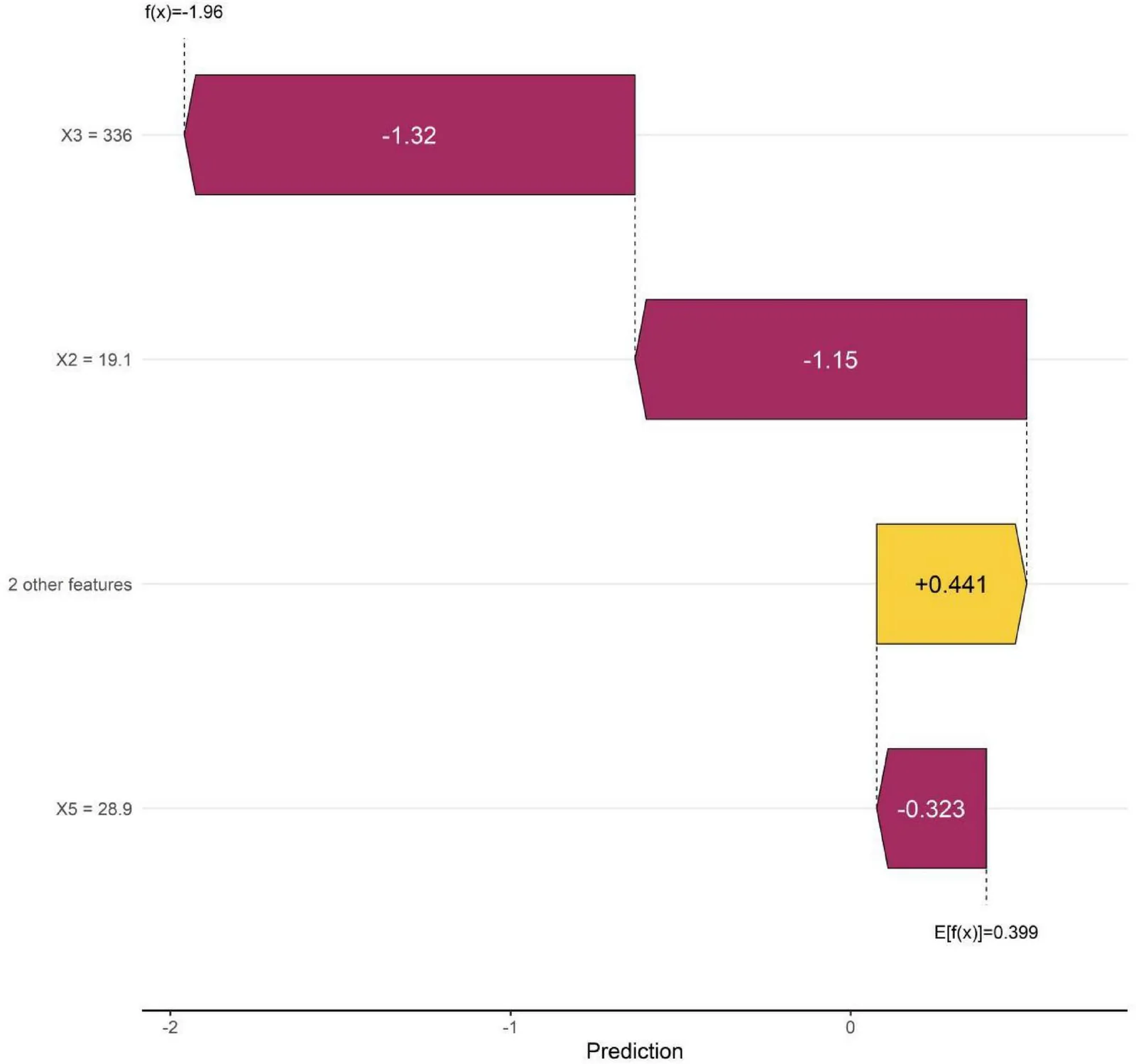
SHapley Additive exPlanations waterfall plot. X1, Admission National Institutes of Health Stroke Scale score; X2, Admission blood glucose; X3, Infarct core volume; X4, Collateral circulation status; X5, Neutrophil-to-lymphocyte ratio.

**FIGURE 6:**
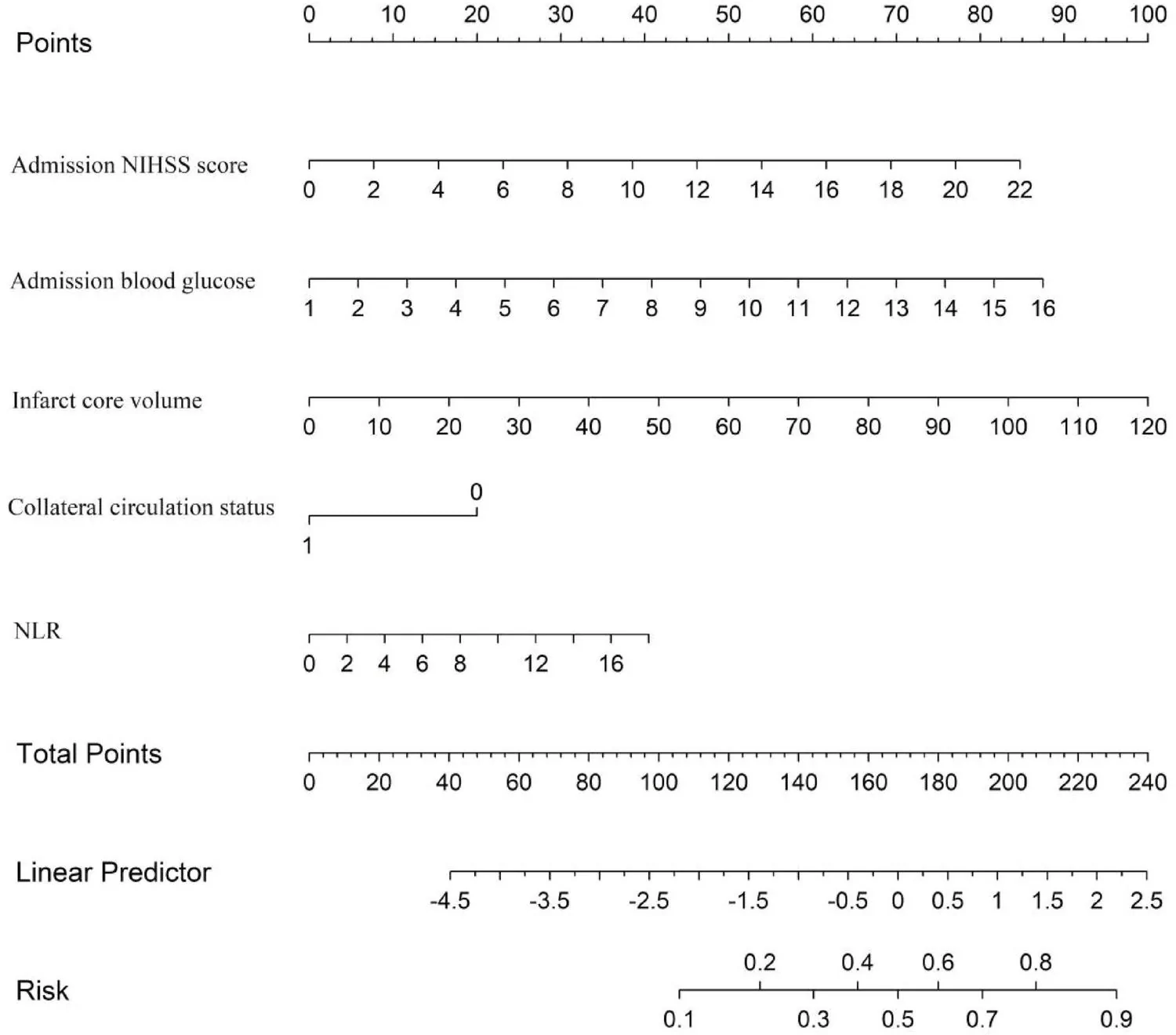
Nomogram for predicting early neurological deterioration risk in acute ischemic stroke patients. NIHSS, National Institutes of Health Stroke Scale; NLR, Neutrophil-to-lymphocyte ratio. Assignment of independent variables (categorical variables): Collateral circulation status (Poor = 0, Good = 1).

Figure 5 presented a case-specific SHAP (Shapley Additive Explanations) waterfall plot, demonstrating the model’s prediction process. Admission NIHSS score, collateral circulation status, and NLR contributed positively to the prediction, whereas admission blood glucose and infarct core volume exerted negative effects. The f (x) value represents the SHAP value for each feature. To facilitate the clinical translation of our model, we further constructed a simple risk score nomogram based on the five core predictors (Figure 6). This nomogram translated the model’s complex algorithm into an intuitive visual tool, allowing clinicians to quickly estimate the probability of END in individual AIS patients by summing the points assigned to each variable and mapping the total score to the corresponding risk probability.

## Discussion

The primary objective of this study was to develop and validate a machine-learning model based on multidimensional data for predicting the risk of END in patients with AIS. In this study, five core predictive indicators were successfully screened from a patient cohort not included in reperfusion therapy: the NIHSS score at admission, infarct core volume, collateral circulation status, -NLR, and blood glucose at admission. A high-performance RF prediction model was then constructed. The AUC values of this model in the training set and validation set were 0.779 and 0.775 respectively, which demonstrated its good discriminatory ability and generalization performance. The five core indicators screened in this study are not isolated risk labels but jointly outline the complex pathophysiological profile of END from different dimensions.

Firstly, the NIHSS score at admission and infarct core volume are two cornerstones for evaluating the severity of the initial stroke insult. The NIHSS score quantifies the degree of neurological deficit from a functional perspective, while the infarct core volume demarcates the extent of irreversible brain damage from an anatomical perspective (14). Together, they constitute the initial environment for the occurrence of END. That is, the more severe the initial injury, the weaker the compensatory reserve of brain tissue. Under the impact of subsequent secondary injury mechanisms, the possibility of functional deterioration is naturally higher. The results of this study confirmed that both indicators were significantly higher in the END group than in the non-END group, which was consistent with the conclusions of numerous previous studies (15–17).

Secondly, the collateral circulation status is a key variable determining the fate of brain tissue after stroke and can be regarded as the “lifeline” of the brain. Good collateral circulation can provide crucial residual blood flow to the ischemic area, especially the ischemic penumbra, through compensatory blood vessels when the main artery is occluded or stenosed (18). This not only helps to delay the expansion of the infarct core but also gains a time window for subsequent possible intervention measures. In this study, collateral circulation was incorporated into the model as a core protective factor, accurately capturing its central position in the fight against END. The data showed that good collateral circulation was a strong protective factor for END, which was completely consistent with the basic physiological role of this indicator.

Thirdly, the roles of systemic inflammatory response and metabolic stress in END were reflected by the NLR and blood glucose at admission. The NLR is a comprehensive inflammatory indicator. Its increase not only reflects the intensification of the pro-inflammatory state dominated by neutrophils but also implies the impairment of the immune regulatory function involving lymphocytes (19). After stroke, the over-activated inflammatory response can attack the ischemic brain tissue, disrupt the blood-brain barrier, induce cerebral edema, and promote microvascular dysfunction, thus directly driving the occurrence of END (20). On the other hand, high blood glucose at admission, whether due to stress response or underlying diabetes, can exacerbate ischemic injury through multiple pathways, including causing intracellular acidosis, increasing oxidative stress levels, and damaging endothelial function (7, 15). The significantly higher NLR and blood glucose levels in the END group in this study strongly supported the important role of systemic inflammation and metabolic disorders in the pathological process of END (21).

Distinctiveness from previous machine learning studies. Several studies have developed machine learning models integrating imaging and clinical information to predict END in AIS (22, 23). However, our study has three key distinctions. First, we explicitly excluded patients receiving reperfusion therapy (intravenous thrombolysis or endovascular thrombectomy) to avoid confounding effects, making our model specifically applicable to AIS patients managed with standard medical therapy—a population that remains substantial in clinical practice, particularly in primary stroke centers or patients presenting beyond the time window for reperfusion. Second, while previous models typically incorporated 2–3 data modalities, our model systematically integrates five core dimensions: functional severity (admission NIHSS), anatomical injury (infarct core volume), cerebrovascular reserve (collateral circulation status), systemic inflammation (NLR), and metabolic stress (admission blood glucose). This multidimensional integration more comprehensively captures the complex pathophysiology of END. Third, we applied SHAP analysis to enhance model interpretability, addressing the “black-box” limitation of machine learning models (24). The SHAP analysis enables visualization of individual feature contributions for each patient, providing clinicians with not only a prediction but also an explanation of the underlying risk drivers.

The excellent performance of the machine-learning algorithm was fully demonstrated in this study. The winning RF algorithm has the advantage of being able to automatically handle complex interactions between variables and is robust to noise and imbalance in the data (25). For example, the protective effect of good collateral circulation may be more significant in patients with a smaller infarct core volume but may be weakened in patients with a huge infarct core. The RF algorithm can well capture such non-linear relationships, which is difficult for traditional linear models. The results also confirmed that the prediction efficacy of the RF model was indeed better than that of traditional methods such as logistic regression.

While ensuring high performance, the model only uses five core indicators. These indicators can be quickly and routinely obtained in most hospitals with stroke units without relying on expensive or complex special examinations. This “less is more” design concept greatly enhances the possibility of promoting and applying the model in real-world clinical settings and avoids the inapplicability of the model due to difficulties in data acquisition.

The successful development of this prediction model provides new tools and ideas for the clinical management of AIS. Its most direct value lies in the ability to achieve early and accurate risk stratification. At the initial stage of patient admission, clinicians can use the model to quickly identify patients at high risk of END. For these high-risk patients, intensive monitoring and management programs can be immediately initiated, such as more frequent neurological function assessments (e.g., once an hour), stricter blood pressure control targets (avoiding too high or too low), active blood glucose management (using an insulin pump to strictly control the blood glucose range), and considering early initiation of intensive statin therapy to reduce inflammation and stabilize plaques (26, 27). This shift from “homogeneous” management to “risk-oriented” individualized and precise management is expected to concentrate medical resources on patients in greatest need, thereby improving the efficiency of prevention and treatment and improving patient outcomes.

In addition, this model lays a foundation for future research. The high-risk population screened based on this model is an ideal target population for clinical trials of new neuroprotective agents or anti-inflammatory drugs. Meanwhile, with the development of mobile healthcare and Internet of Things technologies, the model can be integrated into bedside decision-support systems or mobile terminal apps in the future to achieve dynamic risk assessment and real-time warning, further enhancing the intelligence level of clinical decision-making.

However, this study also has several limitations. Firstly, there are limitations in the study design. This study was a single-center, retrospective design, and patients receiving thrombolysis or thrombectomy were excluded to avoid the confounding effect of reperfusion therapy on END, which inevitably led to selection bias and information bias (28). This exclusion criterion makes the model currently applicable only to AIS patients who do not receive reperfusion therapy, limiting its direct applicability in the whole AIS cohort of modern stroke care that widely adopts reperfusion therapy. Although the model performed well in the internal validation set, its extrapolation performance still needs to be finally confirmed through prospective, multi-center, large-scale external cohort studies. Secondly, the scope of predictive indicators is relatively small. Although the five core indicators in this study cover several important aspects, they still do not include all potential important predictive factors. For example, markers related to thrombus components (such as fibrinogen and dynamic changes in D-dimer) or more specific neuro-injury markers (such as neurofilament light chain protein) were not included (29). Future research can explore incorporating these new biomarkers into the model to further improve the prediction accuracy.

In conclusion, based on the five easily obtainable core clinical indicators of the NIHSS score at admission, infarct core volume, collateral circulation status, NLR, and blood glucose at admission, this study successfully developed and validated a high-performance RF prediction model. This model can effectively identify patients at high risk of early neurological deterioration after AIS, providing a solid and practical tool for early risk stratification and guiding individualized and precise prevention and control, and has important clinical translation value.

## Data Availability

The original contributions presented in this study are included in the article/Supplementary material, further inquiries can be directed to the corresponding author.
