# Supplementary figures and images for: Multidimensional machine learning for early neurological deterioration prediction in acute ischemic stroke
Source: Front Med (Lausanne). 2026 Apr 24;13:1779519. doi: 10.3389/fmed.2026.1779519 (PMC13152818; doi:10.3389/fmed.2026.1779519)

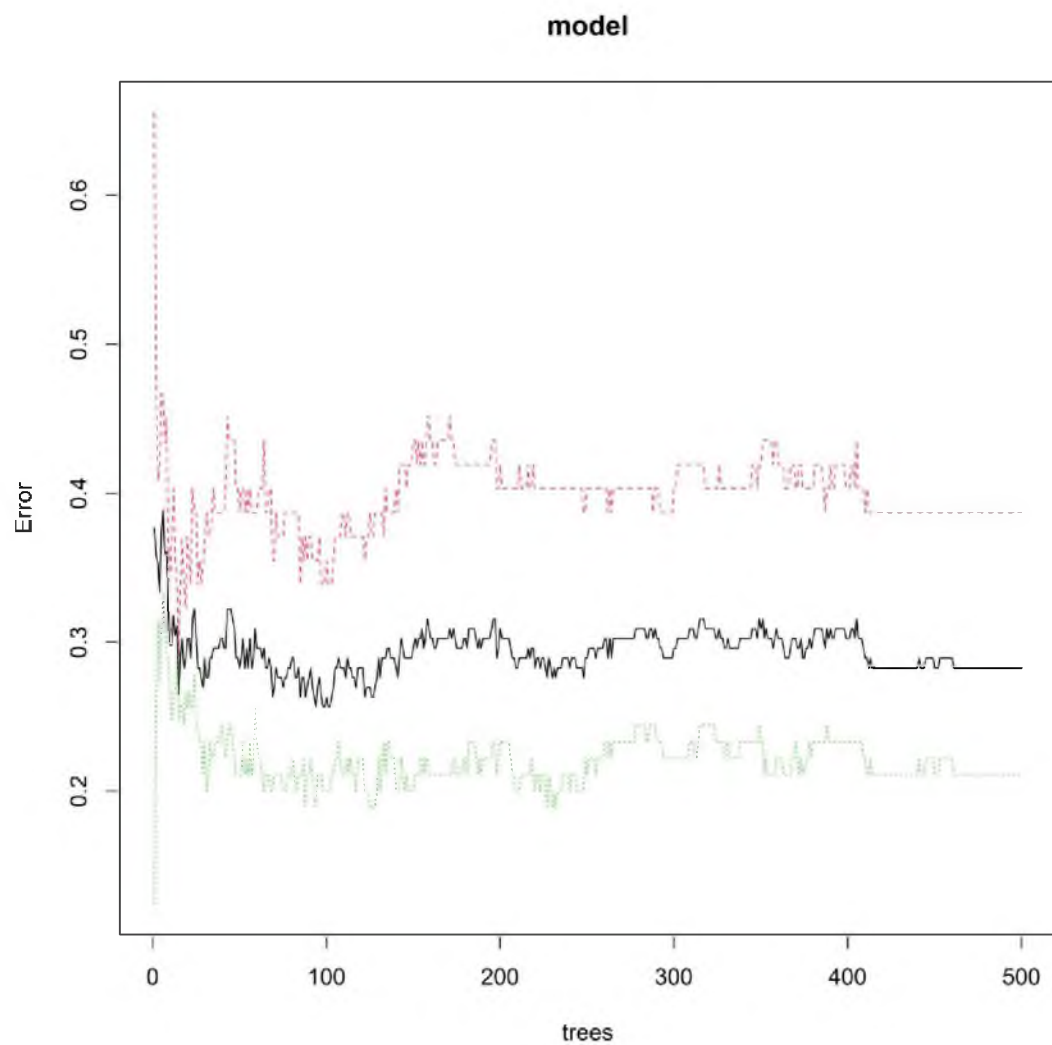

**Supplementary Figure 1. Random Forest model error vs. number of trees.**

Supplement: Supplementary file 1 [file Image_1.pdf]
